# A novel nanobody-based HER2-targeting antibody exhibits potent synergistic antitumor efficacy in trastuzumab-resistant cancer cells

**DOI:** 10.3389/fimmu.2023.1292839

**Published:** 2023-10-25

**Authors:** Xinlin Liu, Linli Luan, Xi Liu, Dingwen Jiang, Junwen Deng, Jiazhen Xu, Yang Yuan, Jiyao Xing, Bingguan Chen, Dongming Xing, Haiming Huang

**Affiliations:** ^1^ The Affiliated Hospital of Qingdao University, Qingdao University, Qingdao, China; ^2^ Qingdao Cancer Institute, Qingdao, China; ^3^ Noventi Biopharmaceuticals Co., Ltd, Shanghai, China; ^4^ Bioworkshops (Suzhou) Limited, Souzhou, China; ^5^ School of Life Sciences, Tsinghua University, Beijing, China

**Keywords:** HER2, nanobody, Fc fusion, trastuzumab-resistance, synergistic efficacy, ligand-dependent heterodimerization

## Abstract

Human epithelial growth factor receptor-2 (HER2) plays an oncogenic role in numerous tumors, including breast, gastric, and various other solid tumors. While anti-HER2 therapies are approved for the treatment of HER2-positive tumors, a necessity persists for creating novel HER2-targeted agents to resolve therapeutic resistance. Utilizing a synthetic nanobody library and affinity maturation, our study identified four anti-HER2 nanobodies that exhibited high affinity and specificity. These nanobodies recognized three distinct epitopes of HER2-ECD. Additionally, we constructed VHH-Fc and discovered that they facilitated superior internalization and showed moderate growth inhibition. Compared to the combination of trastuzumab and pertuzumab, the VHH-Fc combos or their combination with trastuzumab demonstrated greater or comparable antitumor activity in both ligand-independent and ligand-driven tumors. Most remarkably, A9B5-Fc, which targeted domain I of HER2-ECD, displayed significantly enhanced trastuzumab-synergistic antitumor efficacy compared to pertuzumab under trastuzumab-resistant conditions. Our findings offer anti-HER2 nanobodies with high affinity and non-overlapping epitope recognition. The novel nanobody-based HER2-targeted antibody, A9B5-Fc, binding to HER2-ECD I, mediates promising receptor internalization. It possesses the potential to serve as a potent synergistic partner with trastuzumab, contributing to overcoming acquired resistance.

## 1 Introduction

Human epithelial growth factor receptor-2, also known as HER2, is a member of the EGFR family (1). As a receptor tyrosine kinase, overexpressed HER2 is capable of forming homodimers and heterodimers with other members of the EGFR family, thus activating a diverse range of downstream signaling pathways in tumorigenesis. While the specific ligand for HER2 remains unknown, it has been identified as the preferred dimerization partner for other HER proteins, particularly HER3 (2). In addition to enhanced signaling through ligand-dependent heterodimerization, abnormal signaling and ligand-independent dimerization occur with the overexpression or amplification of HER2. Overexpressing-HER2 could evoke tumor-protective antibodies and T cell activation, but these responses are unable to hamper tumor progression (3). The aberrant activation of HER2 receptors has been described in numerous solid tumors, such as breast cancer, gastric cancer, biliary tract cancer, colorectal cancer, ovarian cancer, and pancreatic cancer (1, 4–6). Approximately 20% of breast and gastric cancers showcase HER2 overexpression, which is associated with poor prognosis and an escalated metastatic potential. Consequently, HER2 has merged as a highly attractive therapeutic target.

To date, various types of antibody-based HER2-targeted agents, including monoclonal antibodies and antibody-drug conjugates, have been approved. The first anti-HER2 humanized antibody, trastuzumab, reduces HER2 signaling pathways, prevents cell cycle arrest, and induces antibody-dependent cell-mediated cytotoxicity (ADCC) by recognizing domain IV of the HER2 extracellular domain (ECD) (7, 8). Apart from innate immunity mediated by ADCC, adaptive immune response involving CD8^+^ T cells also contributes to the effective trastuzumab treatment (9). While trastuzumab provides increased clinical outcomes, therapeutic resistance remains a significant challenge. Subsequently, pertuzumab was developed and demonstrated the ability to obstruct HER2 dimerization, courtesy of its binding epitopes anchored in ECD II of HER2 (10). When combined in the preclinical investigation, these two agents exhibited synergistic efficacy. Clinical studies have corroborated that the combined application of trastuzumab and pertuzumab with chemotherapy led to better outcomes for the treatment of HER2-positive metastatic breast cancer (11). However, for the treatment of HER2-positive metastatic gastric or gastro-oesophageal junction cancer, the dual antibodies and chemotherapy failed to surpass trastuzumab plus chemotherapy in terms of overall survival (OS) (12). Another clinical trial demonstrated that the addition of pertuzumab did not enhance the anti-tumor effectiveness of T-DM1 for patients with HER2-positive advanced breast cancer (13). Therefore, there is an urgent need for the development of novel single or combined HER2-targeted therapies to overcome resistance and to maximize clinical benefit.

Ligand-driven HER2-heterodimer formation has been identified as a substantial factor contributing to trastuzumab resistance (14). Although pertuzumab can enhance the inhibitory activity of trastuzumab to some extent, it is challenging to achieve the desired effect in the trastuzumab-resistant tumor. Previous studies suggested that combining trastuzumab with no-competing antibodies could potentially reverse the resistance to ligand-induced proliferation (15–17). Moreover, mixtures of antibodies targeting multiple HER2 subdomains unveiled growth inhibition in cells with acquired resistance to trastuzumab (18). However, the adoption of intricate antibody combinations may augment the risk of immunogenicity, which hampers their further development in clinical practice. Therefore, the quest for an ideal, simplified synergistic partner, to maximize the anti-HER2 efficacy of trastuzumab in resistant tumors, still remains inconclusive.

Nanobodies, also known as single variable domains (VHH), are naturally-derived antigen-binding fragments from heavy-chain only antibodies (HcAbs) of camelids. They have a small molecular weight (~15KDa) and share a highly similar sequence with human VH domains. These features provide nanobodies with lower immunogenicity and the capability to penetrate into tissues more deeply. Moreover, they can withstand high pressures, temperatures, chemical denaturants, and pH values beyond the normal physiological range (19). Compared to other antibody fragments, nanobodies possess a simple structure and are more cost-effective to manufacture. These intrinsic characteristics make nanobodies highly advantageous for cancer treatment applications. The exceptional modularity allows for the engineering and modification of nanobodies to create diverse nanobody-based fusion molecules, such as multi-valent nanobodies, bispecific antibodies, and nanobody-drug conjugates (19). Recent reports have highlighted the antitumor activity of nanobodies or nanobody-based fusions in HER2-positive cancers (20–26). However, their efficacy is limited when it comes to trastuzumab-resistant tumors.

In this study, we successfully obtained four HER2-targeted nanobodies through a synthetic nanobody library. Affinity maturation strategy was applied to improve the affinity of nanobodies. These nanobodies exhibited remarkable antigen-binding specificity and species cross-reactivity. Chimeric HER2-ECD proteins demonstrated that these four nanobodies recognized at least three different domains of HER2-ECD. To further investigate the potential therapeutic benefits, VHH-Fc fusions were constructed and assessed. These fusions were found to induce pronounced receptor internalization and moderate inhibition of tumor cell growth. Interestingly, mixing VHH-Fc or combining them with trastuzumab elicited enhanced antitumor activity. This effect was observed in both ligand-independent and ligand-driven trastuzumab-resistant proliferation scenarios. Among the combinations tested, A9B5-Fc - derived from the nanobody targeting HER2 ECD l - had proven the most robust synergistic potency when combined with trastuzumab. This finding suggests that A9B5-Fc may serve as a more effective therapeutic combination agent for HER2-positive trastuzumab-resistant malignancies.

## 2 Methods

### 2.1 Cell culture, antibodies and reagents

Cell lines were purchased from ATCC (SKBR3 and NCI-N87) and maintained at 37°C/5% CO_2_. Trastuzumab and pertuzumab were produced in-house. A non-specific IgG antibody was used as a negative control and was obtained from Beyotime (A7001).

### 2.2 Phage biopanning

The synthetic nanobody library (ASyNAL) has been established with well quality control (27). A HER2-ECD antigen fused with an Fc tag was used to perform phage biopanning. Briefly, HER2-ECD and Fc protein were coated in two wells of a 96-well microplate overnight, respectively. After 1h incubation with 2% skim milk, the phage library (100 μL/well, ~ 3.0 × 10^12^ phage clones) was added to the Fc wells for 1h incubation to eliminate nonspecific binders. And then the phage solution was transferred to HER2-ECD well with 1h incubation. Next, the well was washed by PT buffer and then the phages were eluted by 100 mM HCl. After the addition of 1 M Tris-HCl for neutralization, the phage solution was used to infect *E.coli* XL1-blue (Stratagene) for 0.5 h. M13KO7 helper phages (NEB, N0315S) were applied for superinfection at a final concentration of 1.0 × 10^10^ phage/mL. Then the solution was cultured in a 2YT medium supplemented with carbenicillin and kanamycin at 32°C. After overnight growth, the XL1-blue culture supernatant was precipitated by PEG/NaCl (20% PEG 8000/2.5 M NaCl) solution. The pelleted phage was reconstituted in a PBS buffer and used as the input phage for the subsequent panning step. In this study, four rounds of panning were applied to enrich the HER2-specific phage particles.

### 2.3 Phage ELISA

HER2-ECD (100 ng/well) and Fc protein (100 ng/well) were coated in wells of a 96-well microplate at 4°C overnight, respectively. After 2% skim milk blocking, the phage solution was introduced for reaction at room temperature for 1 h. PT buffer (300 μL/well) was used to wash the plates at least 8 times and then 50 μL anti-M13/HRP conjugate (Sino Biological) was applied for incubation for 0.5 h. Following PT buffer wash, 50 μL of TMB substrate was added for colordevelopment. After stopping the reaction with 50 μL 1 M H_3_PO_4_, the OD_450_ value of every well was measured by a BioTek plate reader. The data was processed by GraphPad Prism 8.

### 2.4 Affinity maturation library construction

Affinity maturation was performed as previously described (27, 28). Briefly, the pComb3XSS containing HER2-specific nanobodies sequences was applied as a template for library construction. The mutations at CDR regions were incorporated into sequences by the Kunkel method, in which six primers were used to complete the combinatorial reactions. Primer 1 (AGCTGTGCAAGTGGATAAGGATCCTAACTAGGCTGGTTTCGTCAA), Primer 2 (CGCGAAGGAGTTGCTGCATAAGGATCCTAATACTACGCCGATAGCGTG), Primer 3 (CTGTACTATTGTGCGGCCTAAGGATCCTAAAACTACTGGGGCCAAGGC), Primer 4 (AGCTGTGCAGCAAGTGGAN_1_N_1_N_1_N_2_N_1_N_2_N_1_N_4_N_3_N_3_N_1_N_2_N_3_N_2_N_4_N_4_N_4_N_2_N_2_N_2_N_2_CTAGGCTGGTTTCGTCAA), Primer 5 (CGCGAAGGAGTTGCTGCAN_4_N_4_N_2_N _3_N_2_N_4_N_4_N_4_N_2_N_4_N_3_N_1_N_2_N_1_N_2_N_4_N_1_N_1_N_4_N_1_N_2_N_4_N_4_N_2_TACTACGCCGATAGCGTG), and Primer 6 (CTGTACTATTGTGCGGCCN_3_N_2_N_4_N_4_N_2_N_2_N_4_N_2_N_2_N_2_N_2_N_2_N_1_N_4_N_3_N_3_N_4_N_2_N_4_N_1_N_2_N_1_N_4_N_3_N_3_N_3_N_4_N_4_N_1_N_1_N_2_N_2_N_2_N_3_N_2_N_4_N_4_N_3_N_1_N_3_N_1_N_4_AACTACTGGGGCCAAGGC). Next, double-strand DNA (dsDNA) was digested by BamH I and was transfected into SS320. Bacterial serial dilution was used to confirm the library diversity.

### 2.5 Protein expression and purification

The DNA sequence encoding nanobodies were cloned into expression vector pET22b followed by C-terminal 6 × His tag via One Step Cloning Kit (Vazyme, C112). The nanobody’s expression and purification were performed as previously described (27). Briefly, the vectors were transformed into BL21 (DE3). The nanobodies expression was induced by 0.2 mM IPTG at 18°C overnight. The pellets were collected and treated with a lysis buffer. Next, the lysate was heated at 60°C for 0.5 h to remove the denatured proteins. The supernatant was subjected to Ni Bestarose FF (BestChrom, AA0051) to purify the nanobodies. The final purification was buffer exchanged into PBS.

The DNA sequence encoding nanobodies were cloned into pSCSTa followed by a C-terminal Fc tag. The nanobody Fc fusions (VHH-Fc) were expressed from Expi 293 cells. The pSCSTa was transiently transfected into 293 cells. After 120 h culture, the supernatant was collected and then purified by Protein A affinity chromatography using AT Protein A Diamond Plus (BestChrom, AA402305). The eluted antibodies were dissolved in PBS buffer. The concentrations of all purified proteins were confirmed by BCA method and their purity was analyzed on SDS-PAGE gels.

### 2.6 Biolayer interferometry (BLI)

The BLI assay was performed as previously described (27). Briefly, the nanobodies with His tag were immobilized on the surface of the Ni-NTA biosensor. The HER2-ECD with Fc tag at different concentrations was used to measure association and dissociation. The response data was recorded by Octet RED96 System (ForteBio) and was analyzed by Octet software.

### 2.7 ELISA

For indirect ELISA, the 96-well plates were coated with HER2-ECD or other related antigens in PBS buffer at 4°C overnight. After 2% skim milk blocking at room temperature for 2 h, the series of diluted antibodies were added to wells at room temperature for 1 h. The HRP-conjugated secondary antibodies (GenScript, A01861) were incubated at room temperature for 0.5 h. Following PT buffer wash, TMB substrate was added for color reaction. After stopping the reaction with 1 M H_3_PO_4_, the OD_450_ value of every well was measured by a BioTek plate reader.

For the competitive protein ELISA, HER2-ECD was coated in the 96-well plates. After 2% skim milk blocking at room temperature for 2 h, the sub-saturation concentration of nanobodies (His tag) was mixed with a series of diluted VHH-Fc or other antibodies. Next, the mixture was added to react with HER2-ECD at room temperature for 1 h. The HRP-conjugated mouse anti-human IgG Fc antibody (GenScript, A01854) was used as a secondary antibody. After stopping the reaction with 1 M H_3_PO_4_, the OD_450_ value of every well was measured by a BioTek plate reader. The ELISA data was processed by GraphPad Prism 8.

### 2.8 Cell-surface binding by flow cytometry

HER2-expressing tumor cells were resuspended in PBS + 2% FBS and planted in 96-well plates at 5 × 10^4^ cells/well. The cells were incubated with diluted HER2-specific antibodies at 4°C for 1 h. After washing the unbound antibodies, cells were treated with PE-labeled anti-huFc (Abcam, 98596) secondary antibody (1:1000) at 4°C for 0.5 h. The cells were resuspended by 120 μL PBS + 2% FBS. Beckman Coulter flow cytometer was used to collect the mean fluorescent intensity (MFI) values from the secondary antibody. The binding data was calculated by GraphPad Prism 8.

### 2.9 Confocal imaging

NCI-N87 cells settled onto coverslips at 37°C/5% CO_2_ overnight. Then, 50 nM antibodies were added to incubate with cells at 4°C for 1 h. The cells were washed to remove unbound antibodies and then were fixed with 4% paraformaldehyde (PFA) at room temperature for 20 min. Triton X-100 was used to permeabilize the cells. Cells were treated with goat anti-human IgG Fc (DyLight^®^ 488) to detect the antibody-receptor complex (green), and anti-LAMP1 antibody (Abcam, ab25630) followed by Alexa-Fluor 647-labeled goat anti-mouse IgG H&L (Abcam, ab150115) to stain the lysosomes (red). Cells were next stained by DAPI (blue) and coverslipped with an antifade mounting medium (Beyotime, P0126). Imaging was performed by Nikon A1 confocal microscope and was analyzed by NIS elements viewer software.

### 2.10 Epitope mapping

The DNA sequences encoding chimeric HER2-ECD proteins were constructed by replacing the ECD I (T23-R217), ECD II (T218-C342), ECD III (Y344-A510), and ECD IV (C511-T652) with those of murine homolog, respectively. The chimeric HER2-ECD proteins (HER2-mD1, HER2-mD2, HER2-mD3, and HER2-mD4) were cloned into a pSCSTa vector with a C-terminal Fc tag and expressed by 293T cells. The methods of expression and purification of chimeric proteins were the same as that of VHH-Fc. The purity of proteins was resolved by 10% SDS-PAGE.

To identify the binding epitopes of HER2-specific nanobodies, the 96-well plates were coated with chimeric or wild-type HER2-ECD proteins in PBS buffer at 4°C overnight. After 2% skim milk blocking at room temperature for 2 h, the series of diluted nanobodies (His tag) or antibodies were added to wells at room temperature for 1 h. The HRP-conjugated His-Tag monoclonal antibody (Proteintech, HRP-66005) was used to detect the nanobodies and the HRP-conjugated mouse anti-human IgG Fab antibody (GenScript, A01855) was applied for binding to IgG antibodies. After incubation at room temperature for 0.5 h, the subsequent experiments were performed as above described.

### 2.11 Internalization

HER2-expressing tumor cells were treated with HER2-specific antibodies on ice for 1 h. After washing with PBS + 2% FBS, an aliquot of cells was kept on ice, while the remainder was cultured in an incubator for 0.5 h or 4 h. The frozen 4% paraformaldehyde was used to fix cells for 15 min. Cells were then incubated with PE-labeled anti-huFc (Abcam, 98596) secondary antibody (1:1000) at 4°C for 30 min and measured by flow cytometry. The HER2 internalization rate was estimated as a percentage MFI loss at 37°C relative to that on ice.

### 2.12 Growth inhibition

Tumor cells were seeded in 96-well plates at 37°C/5% CO_2_ overnight. The diluted antibodies (1:5 serial dilution) were added and incubated with cells for 5 days. Cell Counting Kit-8 (CCK-8) was used to determine cell viability. Briefly, the supernatant in wells was removed and cells were treated with a culture medium with 10% cck8 at 37°C/5% CO_2_ for 2h. The OD_450_ value was measured by a BioTek plate reader. For ligand-dependent growth inhibition, the 5 nM EGF (SinoBiological, GMP-10605-HNAE) or 1 nM HRG (SinoBiological, 11609-HNCH) were added to stimulate tumor cells. The data and p-values were analyzed by GraphPad Prism 8.

## 3 Results

### 3.1 Screening the HER2-ECD specific nanobodies from a VHH phage display library

**Figure 1:**
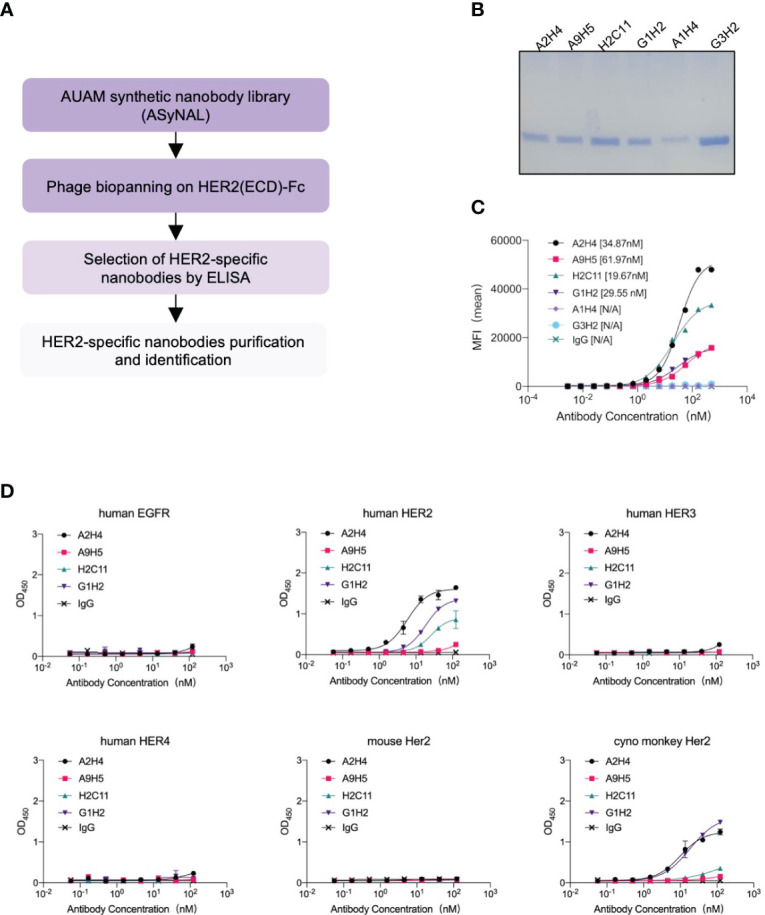
Screening and characterization of HER2-specific nanobodies. **(A)** The flowchart summarized the overall process of nanobodies screening from the AUAM synthetic nanobody library (ASyNAL). **(B)** HER2-specific nanobodies with His-tag were purified from BL21 (DE3) and showed high purity in SDS-PAGE. **(C)** The cell-surface binding activity of nanobodies was quantified by flow cytometry in SKBR3 tumor cells. **(D)** Nanobodies showed target specificity and species cross-reactivity. ELISA assay was performed to analyze the binding of nanobodies against human EGFR, human HER2, human HER3, human HER4, mouse Her2, and monkey Her2. In c and d, a non-specific IgG antibody was used as a negative control.

A synthetic nanobody library, named AUAM synthetic nanobody library (ASyNAL) has been constructed in our previous studies (27). Through this library, we have successfully obtained nanobodies for diverse antigens including SH2 domain of tyrosine-protein kinase Fyn (Fyn_SH2), glutathione S-transferase (GST), and human SARS-CoV-2 spike protein (27–29). To generate HER2-specific nanobodies, we conducted panning of ASyNAL against recombinant HER2-ECD protein (**Figure 1A**). Using phage ELISA, we identified six HER2-specific nanobodies, namely A2H4, A9H5, H2C11, G1H2, A1H4, and G3H2 (**Figure 1B**). We next assessed the cell surface binding of these nanobodies to HER2-over-expressing SKBR3 cells by flow cytometry (**Figure 1C**). The results showed that G3H2 and A1H4 did not bind to SKBR3 cells, while the other four nanobodies (A2H4, A9H5, H2C11, and G1H2) exhibited moderate binding activities with EC_50_ values ranged from 19.67-61.97 nM. To confirm the specificity of nanobodies for HER2 binding, we used an ELISA assay to determine their binding activity against various recombinant proteins, including human EGFR, human HER2, human HER3, human HER4, mouse Her2, and monkey Her2 (**Figure 1D**). The results indicated that these four nanobodies exhibited a clear specificity for HER2 binding and also demonstrated species cross-reactivity. To further investigate the binding affinities of these nanobodies to HER2 antigens, we employed biolayer interferometry (BLI) and obtained KD values ranging from 98.13-750 nM (**Table 1**). Considering the relatively low binding affinity to HER2 proteins, we aimed to engineer these four HER2-specific nanobodies by phage display to improve their affinities.

**Table 1:** The affinities of anti-HER2 nanobodies binding to HER2-ECD.

	Affinity		
Anti-HER2 nanobodies	KD (nM)	kon(1/Ms)	kdis(1/s)
A2H4	98.13	2.51 × 10^4^	2.47 × 10^-3^
A9H5	136.7	2.71 × 10^4^	3.7 × 10^-3^
H2C11	509.5	9.12 × 10^4^	4.65 × 10^-2^
G1H2	750	3.88 × 10^4^	2.91 × 10^-2^

### 3.2 Affinity maturation of HER2-targeted nanobodies by phage display

**Figure 2:**
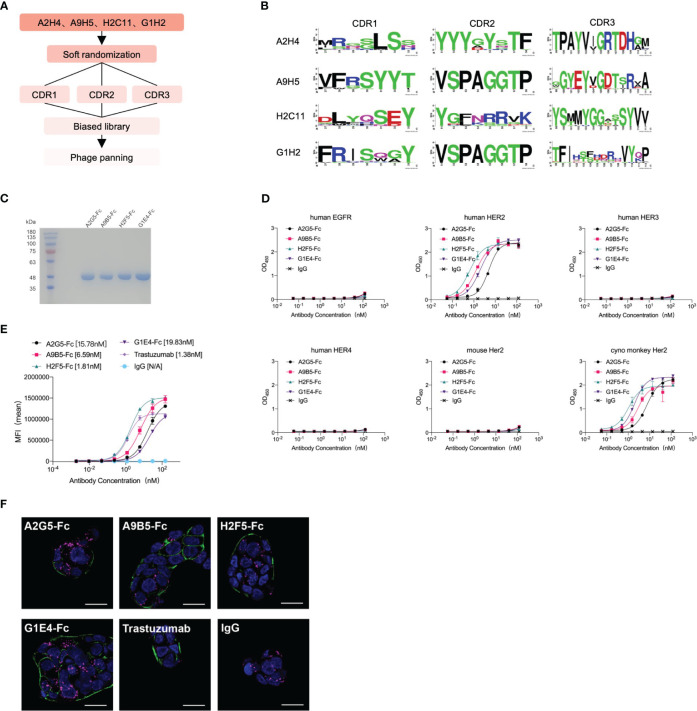
Affinity maturation of HER2-targeted nanobodies by phage display and identification of VHH-Fc. **(A)** The flowchart summarized the overall process of affinity maturation. **(B)** The sequence alignments of CDR regions were represented by Weblogo (http://weblogo.berkeley.edu/logo.cgi). All CDR regions of A2H4 and H2C11 were designed to generate random mutations. As for A9H5 and G1H2, their CDR2 region remained unchanged. **(C)** VHH-Fc fusions were purified from the 293T system and resolved by SDS-PAGE. **(D)** VHH-Fc derived from HER2-nanobodies with high affinity retained target specificity and species cross-reactivity. VHH-Fc showed a potent binding activity to human HER2 and monkey Her2 recombinant proteins. **(E)** VHH-Fc exhibited greater saturation activity in tumor cells compared to related parental nanobodies. **(F)** Confocal images of NCI-N87 cells identified the binding of VHH-Fc to the cell surface. Cells were treated with 50 nM antibodies and were stained by DyLight^®^ 488 for detection of the HER2-antibody complex. Alexa-Fluor 647-labeled secondary antibody and DAPI were used to visualize lysosomes and nuclei, respectively. Scale bars = 10 μm.

We employed a technique called “soft randomization” to enhance the binding affinity of four nanobodies (A2H4, A9H5, H2C11, G1H2) against HER2 (**Figure 2A**). The Kunkel approach was utilized to concurrently randomize two or three CDRs of nanobodies and the resulting biased library was described in the Method section. After multiple rounds of phage ELISA panning, we obtained four groups of variants that selectively bind to HER2. The multi-alignment data clearly displayed the variable residues and conserved residues within the CDRs (**Figure 2B**). For A2H4 and H2C11, we simultaneously randomized three CDR regions, while for A9H5 and G1H2, we randomized CDR1 and CDR3 accordingly. Notably, the variable residues of CDR3 from all four nanobodies were mainly distributed at the end of the loop. In addition to the areas in the middle section, this highly variable region may allow CDR3 to evolve additional binding capability.

Two variants in each group were randomly selected for expression and purification. The BLI data revealed that the binding affinity of HER2-specific nanobodies was significantly improved through affinity maturation (**Table 2**). Due to the decreased Kdis value, the binding affinity of variants to HER2 has got about five-fold or even a hundred-fold increase than that of the original nanobodies. The variants (A2H4-G5, A9H5-B5, H2C11-F5, and G1H2-E4; hereafter referred to as A2G5, A9B5, H2F5, and G1E4) with top affinity in each group were selected for further investigations. To ascertain the functions of these HER2-specific nanobodies, they were fused with human IgG1 Fc to construct VHH-Fc antibodies (A2G5-Fc, A9B5-Fc, H2F5-Fc, and G1E4-Fc), respectively. These fusion antibodies were expressed in the 293T system and purified by ProteinA affinity chromatography. The SDS-PAGE showed that the purity of each VHH-Fc exceeded 95% (**Figure 2C**). Given that affinity maturation might compromise the binding specificity, we used the aforementioned ELISA method to evaluate the antigen-binding activity of VHH-Fc. Results demonstrated that the variants retained binding specificity toward HER2 and also displayed cross-reactivity across species. Compared to relative parental nanobodies, VHH-Fc showed significantly higher binding activity to human HER2 and cyno-monkey HER2 (**Figure 2D**).

**Table 2:** The affinities of anti-HER2 nanobodies binding to HER2-ECD after affinity maturation.

	Affinity		
Anti-HER2 nanobodies	KD (nM)	kon(1/Ms)	kdis(1/s)
A2H4-H3	42.17	3.16 × 10^4^	1.33 × 10^-3^
A2H4-G5	14.37	5.11 × 10^4^	7.34 × 10^-4^
A9H5-F5	19.38	2.76 × 10^4^	5.35 × 10^-4^
A9H5-B5	7.801	5.93 × 10^4^	4.63 × 10^-4^
H2C11-A6	7.25	4.59 × 10^4^	3.32 × 10^-4^
H2C11-F5	3.89	9.87 × 10^4^	3.84 × 10^-4^
G1H2-H4	88.3	3.82 × 10^4^	3.37 × 10^-3^
G1H2-E4	53.4	3.64 × 10^4^	1.94 × 10^-3^

To exam the enhanced cell surface binding of nanobodies after affinity maturation and Fc domain fusion, we tested the cell saturation of VHH-Fc compared to trastuzumab via flow cytometry in a HER2-expressing tumor cell NCI-N87 (**Figure 2E**). The EC_50_ values of VHH-Fc ranged from 1.81-19.83 nM and H2F5-Fc showed the highest binding activity, due to its potent affinity to HER2 (**Table 2**). To further confirm the cell surface binding, we treated NCI-N87 cells with different VHH-Fc and then observe the cell surface fluorescent signals by confocal microscopy. As shown in **Figure 2F**, all four VHH-Fc exhibited comparable strong staining on the surface of HER2-positive cells with trastuzumab.

### 3.3 Epitopes mapping of HER2-ECD specific nanobodies

**Figure 3:**
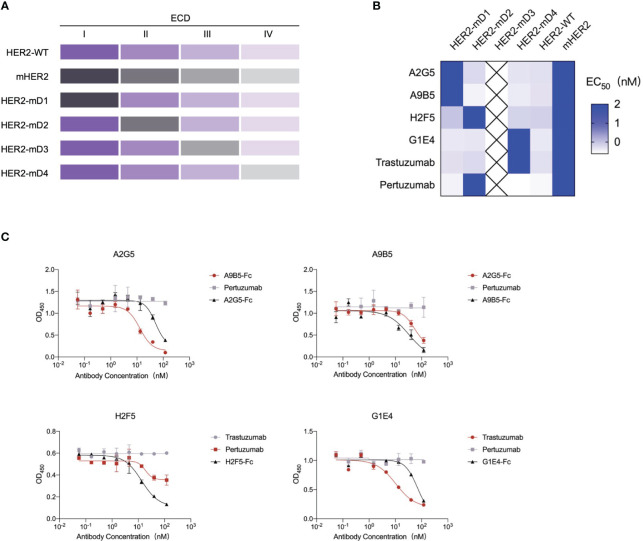
Epitope analysis of HER2-targeted nanobodies. **(A)** Schematic diagram of the construction of chimeric HER2-ECD proteins with domain substitution. HER2-mD1, HER2-mD2, HER2-mD3, and HER2-mD4 corresponded to the ECD I (T23-R217), ECD II (T218-C342), ECD III (Y344-A510), and ECD IV (C511-T652) of HER2 replaced by that of murine homolog, respectively. All chimeric and wild-type HER2 proteins were expressed in the 293T system. **(B)** ELISA analysis of the key domains of interactions between HER2 and nanobodies. The EC_50_ value of nanobodies against different recombinant HER2 proteins was calculated and displayed by a heat map. **(C)** Competitive ELISA confirmed the binding domains of nanobodies. A2G5 and A9B5 competed with each other to bind HER2. H2F5 partially competed with pertuzumab, and G1E4 competed with trastuzumab.

There are four domains in the extracellular regions of HER2 and distinct binding regions may induce various functions of antibodies (7, 16, 30). To identify the epitope diversity, we substituted each of the four domains of HER2 with those of a mouse homolog to construct four chimeric HER2-ECD proteins (HER2-mD1, HER2-mD2, HER2-mD3, and HER2-mD4), which were fused to human IgG1 Fc and then expressed in the 293T system (**Figure 3A**). Because of the extremely low expression of HER2-mD3, the remaining three chimeric proteins, along with two wild-type recombinant HER2 proteins were used to measure the binding epitopes of nanobodies by ELISA (**Figure 3B**). The results showed that only HER2-mD1 reduced the binding activity of A2G5 and A9B5, suggesting that their binding regions were located on the domain I of human HER2. Similar to pertuzumab, H2F5 recognized the domain II of HER2. The diminished binding activity of G1E4 toward HER2-mD4 suggested the binding epitope of G1E4 was involved in domain IV, which is identical to trastuzumab.

A competition ELISA was conducted to validate the above mapping results (**Figure 3C**). A2G5 and A9B5 exhibited mutual blockade, demonstrating that they could bind to overlapping regions in domain I of HER2-ECD. The binding activity of H2F5 was reduced by pertuzumab but not trastuzumab, suggesting that H2F5 and pertuzumab recognized overlapping epitopes of domain II. Trastuzumab provided a stronger blockade for G1E4 binding compared to its own control, signifying that the epitopes recognized by G1E4 were included in those recognized by trastuzumab. Taken together, our analysis revealed that these four anti-HER2 nanobodies could recognize at least three different epitopes (ECD I, ECD II, and ECD IV), respectively.

### 3.4 HER2-targeted VHH-Fc promotes receptor internalization and further inhibits tumor cell growth

**Figure 4:**
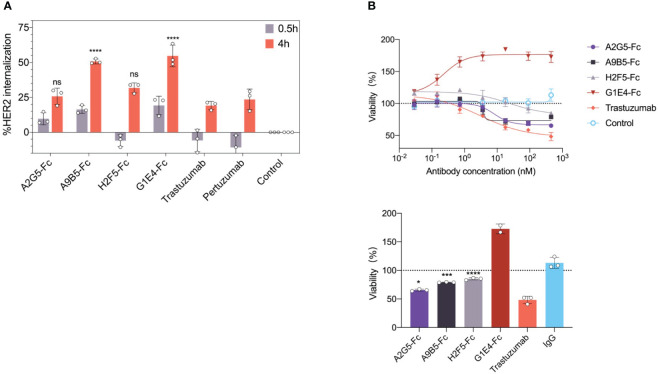
HER2-targeted VHH-Fc promotes internalization and mediates growth inhibition in SKBR3 and NCI-N87 tumor cells. **(A)** HER2 internalization induced by VHH-Fc in comparison with trastuzumab and pertuzumab. SKBR3 cells were incubated with antibodies for 0.5 or 4 hours. The flow cytometry was used to determine the mean percent internalization. Data were mean ± SD (n=3) and two-way ANOVA with Tukey’s multiple comparisons test was performed. The p values were provided in **Supplementary Table 1**. **(B)** VHH-Fc mediated tumor growth inhibition in NCI-N87 cells. The percentage of cell viability was measured by CCK-8 assay. (Up) The representative curve means the concentration-dependent inhibition induced by VHH-Fc. (Down) A2G5-Fc, A9B5-Fc, and H2F5-Fc conferred moderate cell proliferation blocking, but G1E4-Fc showed significant agonistic effect. Data were mean ± SD (n=3) and one-way ANOVA with Dunnett’s multiple comparisons test was performed. The p values were provided in **Supplementary Table 2**. *p < 0.0332, ***p < 0.0002, ****p < 0.0001.

It has been reported that some antibodies can promote cell surface receptor clustering, resulting in receptor internalization and subsequent lysosomal degradation (31). We investigated HER2 internalization in SKBR3 cells by the treatment of four VHH-Fc at 0.5 h or 4 h. The analysis of flow cytometry showed that A9B5-Fc and G1E4-Fc both were able to induce significantly higher internalization rates than trastuzumab or pertuzumab at 4h (**Figure 4A**; **Supplementary Table 1**). A2G5-Fc and H2F5-Fc provided comparable receptor internalization rates to trastuzumab or pertuzumab.

To determine whether HER2 internalization would also inhibit cell growth by our nanobodies, we assessed the effects of VHH-Fc on NCI-N87 cells which have high HER2 expression. A pattern of concentration-dependent inhibition of cell viability was observed following the introduction of A2G5-Fc, A9B5-Fc, and H2F5-Fc (**Figure 4B**). However, their effects were lower than that of trastuzumab (**Supplementary Table 2**). Surprisingly, G1E4-Fc mediated a significant promotion of cell viability (**Figure 4B**). In summary, A9B5-Fc was the sole candidate that managed to promote both HER2 internalization and tumor cell growth inhibition.

### 3.5 The combinations of VHH-Fc inhibit ligand-independent and ligand-driven proliferation of HER2-positive cancer cells with potent synergistic efficacy

**Figure 5:**
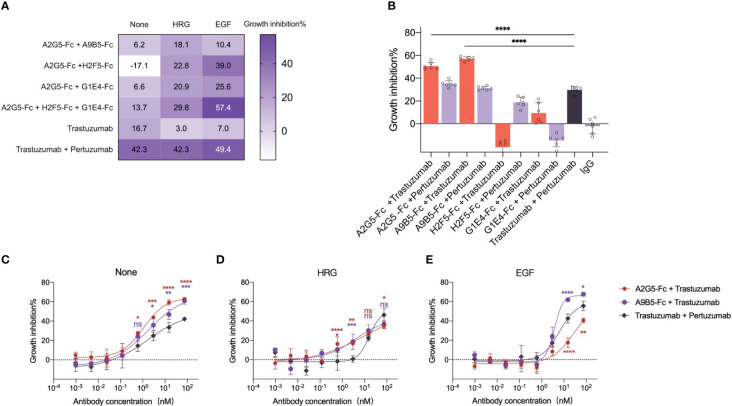
VHH-Fc exhibits potent synergistic efficacy with antibodies recognizing non-overlapping epitopes irrespective of the presence of ligands. **(A)** VHH-Fc mixtures mediated the inhibition of ligand-dependent growth in NCI-N87 cells. A heat map was used to represent the inhibitory rate (mean, n=3, two-way ANOVA with Dunnett’s multiple comparisons test). The p values were provided in **Supplementary Table 3**. **(B)** VHH-Fc showed greater synergistic efficacy with trastuzumab than a combination of trastuzumab and pertuzumab. A2G5-Fc and A9B5-Fc, derived from nanobodies binding to HER2 ECD I, both induced a significantly greater combined effect with trastuzumab than pertuzumab. Data were mean ± SD (n=6) and one-way ANOVA with Dunnett’s multiple comparisons test was performed. The p values were provided in **Supplementary Table 4**. **(C-E)** A9B5-Fc mediated superior synergistic efficacy with trastuzumab to block ligand-independent and ligand-driven tumor cell growth. Data were mean ± SD (n=3) and two-way ANOVA with Dunnett’s multiple comparisons test was performed. The p values were provided in **Supplementary Table 5**. *p < 0.0332, **p < 0.0021, ***p < 0.0002, ****p < 0.0001.

It has been reported that the combination of two or more antibodies targeting non-overlapping epitopes achieved synergistic anticancer activity in preclinical and clinical studies (17, 18, 32–36). To investigate this phenomenon, we confirmed that four HER2-targeted nanobodies with high affinity were able to bind three different epitopes of HER2-ECD (**Figure 3B**). Then we evaluated the synergistic inhibition effects of the combination of nanobody-based antibodies (VHH-Fc) in HER2-expressing tumor cells. NCI-N87 cells were treated with trastuzumab, trastuzumab plus pertuzumab, and VHH-Fc mixtures. The results showed that the inhibitive impact of the VHH-Fc mixtures was on par with trastuzumab alone, but underwent a decline when compared with the combination of trastuzumab with pertuzumab (**Figure 5A** and **Supplementary Table 3**). Intriguingly, the combination of A2G5-Fc and H2F5-Fc led to agonistic behavior, in contrast to the inhibitive function that each had on cell growth when independently administered (**Figure 4B**).

Ligand-driven HER2-heterodimer formation caused compensatory signaling and proliferation, a process contributing to trastuzumab resistance (37, 38). The ability of VHH-Fc combinations to combat acquired resistance was evaluated in NCI-N87 in the presence of epidermal growth factor (EGF) or heregulin (HRG). The trastuzumab resistance was induced by ligands addition. Obviously, trastuzumab exhibited virtually no activity in ligand-stimulating tumor cells, manifesting tangible growth inhibition only upon combination with pertuzumab (**Figure 5A**). In contrast, all VHH-Fc mixtures showed a superior inhibitory impact than trastuzumab under the condition of HRG-driven cell growth (**Figure 5A**). In the EGF-stimulated context, the tripartite mixture (A2G5-Fc + H2F5-Fc + G1E4-Fc) or a paring of A2G5-Fc and H2F5-Fc demonstrated an inhibitory effect comparable to the combination of trastuzumab and pertuzumab (**Figure 5A** and **Supplementary Table 3**). In general, specific VHH-Fc mixtures realized efficacy comparable to a trastuzumab and pertuzumab combination in trastuzumab-resistant tumor cells.

Further investigation was conducted to evaluate the collaborative efficacy of VHH-Fc in combination with trastuzumab or pertuzumab. As delineated in **Figure 5B**, A2G5-Fc and A9B5-Fc presented significantly higher synergistic growth inhibitory activity when combined with trastuzumab or pertuzumab, compared to the remaining VHH-Fc. Specifically, combinations of trastuzumab with A2G5-Fc or A9B5-Fc resulted in an approximately 20% increase in inhibition rate compared to the combination of trastuzumab and pertuzumab (**Supplementary Table 4**). Meanwhile, combinations of pertuzumab with A2G5-Fc or A9B5-Fc mediated comparable suppression of cell growth to trastuzumab plus pertuzumab (**Supplementary Table 4**). In contrast, when combined with trastuzumab or pertuzumab, H2F5-Fc and G1E4-Fc exhibited limited synergistic effects and, rather worryingly, promoted further tumor cell proliferation (**Figure 5B**). These data underlined that A2G5-Fc and A9B5-Fc, derived from nanobodies targeting ECD I, proofed to be superior synergistic partners with trastuzumab in comparison to pertuzumab.

We subsequently undertook a critical analysis whether this synergistic anti-tumor activity works in cells demonstrating trastuzumab resistance induced by adding the ligand. Combinations of trastuzumab with A2G5-Fc or A9B5-Fc had concentration-dependent inhibitory activity in NCI-N87 cells, irrespective of the presence of ligands. Consistent with the above results (**Figure 5B**), A2G5-Fc and A9B5-Fc both showed significantly greater synergistic efficacy with trastuzumab than pertuzumab in ligand-independent tumor cells (**Figure 5C**). The combined effect of trastuzumab and pertuzumab was reduced by the addition of HRG, revealing growth-inhibitory activity at higher concentrations (75nM and 15nM) (**Figure 5D**). Notably, administering A9B5-Fc with trastuzumab demonstrated comparable efficacy to the trastuzumab-pertuzumab paring in higher concentrations; however, it exhibited a markedly higher rate of inhibition in lower concentrations (3nM and 0.6nM). Furthermore, the combination of A9B5-Fc and trastuzumab clearly outperformed trastuzumab plus pertuzumab when it came to hindering the growth of EGF-driven cells (**Figure 5E**). These data unequivocally demonstrated that the combined efficacy of A9B5-Fc and trastuzumab was greater or comparable to the trastuzumab-pertuzumab paring in treatment of ligand-independent and ligand-driven tumor cells. In a word, A9B5-Fc was a more effective synergistic partner for trastuzumab than pertuzumab in trastuzumab-resistant tumors.

## 4 Discussion

HER2-targeted therapies have led to marked improvement in survival outcomes. Nevertheless, given that not all patients diagnosed with HER2-positive cancer reaps the benefit of these treatments, compelling urgency infuses the mission to develop even more proficient anti-HER2 strategies (39). Our research has paved a pathway toward the discovery and refinement HER2-specific nanobodies that demonstrate high affinity along with a promising level of internalization activity, courtesy of the AUAM synthetic nanobody library (ASyNAL) – an instrument previously employed in the successful creation of potent nanobodies for a range of antigens (27).

**Figure 6:**
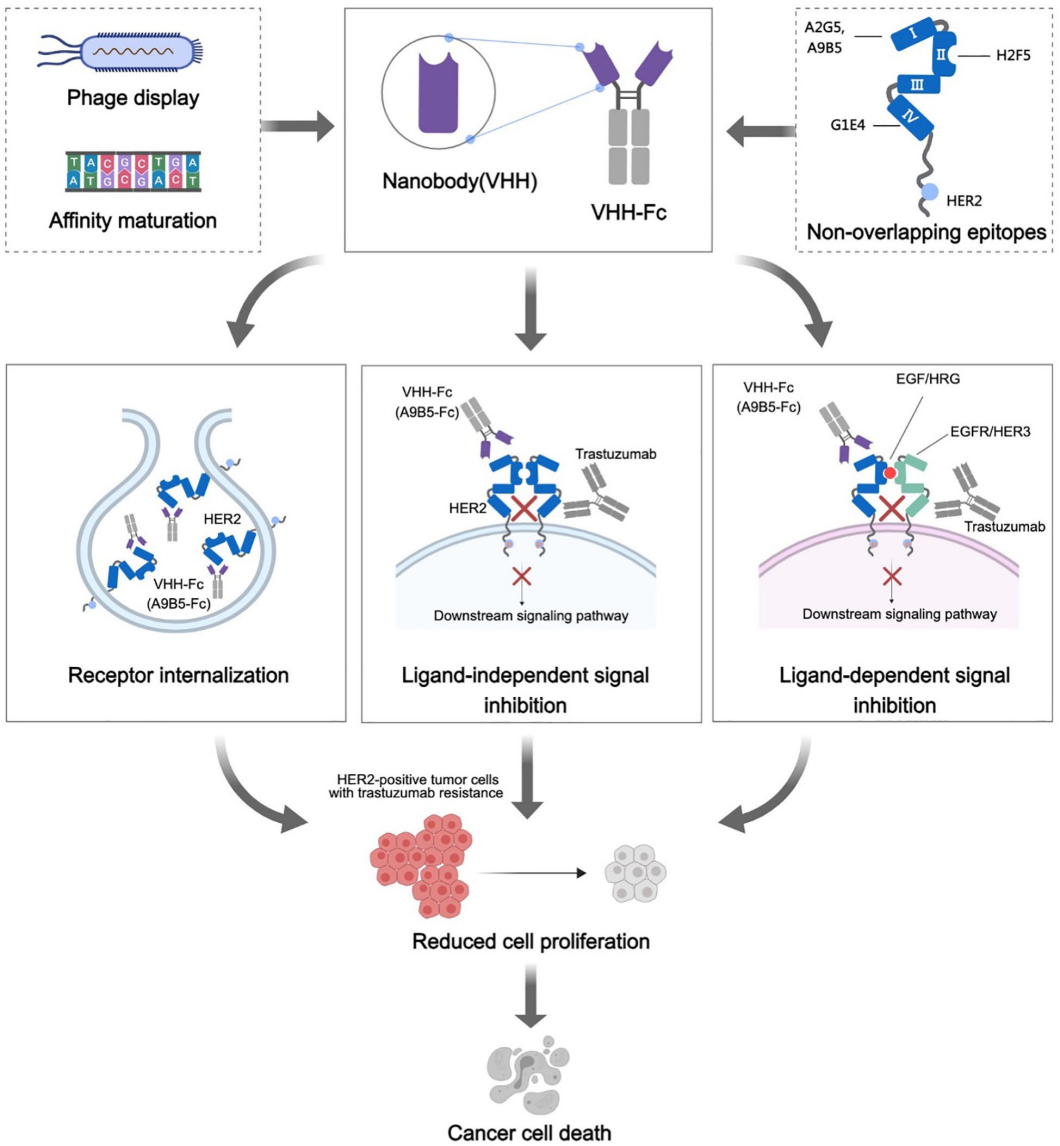
Mechanisms of action of HER2-targeted VHH-Fc. HER2-specific nanobodies were obtained from a well-established synthetic nanobody library (ASyNAL). Affinity maturation based on soft randomization led to a pronounced increase in nanobodies binding to HER2 receptors of tumor cells. These nanobodies could recognize at least three different epitopes, encompassing ECD I, ECD II, and ECD IV. We further constructed the VHH-Fc fusions, which theoretically could prolong the plasma half-life of VHH, improve therapeutic benefit by bivalent binding, and induce Fc-effector functions (ADCC and CDC). The HER2 receptor internalization induced by VHH-Fc was notably observed in HER2-positive tumor cells, indicative of VHH-Fc potentially mediating receptor clustering on the cell surface. A9B5-Fc, targeting ECD I, exhibited potent combined efficacy with trastuzumab in ligand-independent and EGF/HRG-stimulating tumor cell growth. We posited that a combination of A9F5-Fc and trastuzumab effectively disturbed HER2 heterodimerization and homodimerization with EGFR family members, resulting in a blockade of ligand-independent and ligand-driven resistance. In summary, these mechanisms contribute to the growth suppression of trastuzumab-resistant tumor cells by the novel nanobody-based VHH-Fc.

Our findings underpin the premise that these HER2-targeted nanobodies showcase an elevated binding propensity toward HER2-expressing tumor cells, while preserving species cross-reactivity. By meticulously designing chimeric HER2-ECD proteins, we ascertained that these four nanobodies recognized a minimum of three discrete yet complimentary epitopes (ECD I, II, and IV). Based on this, we hypothesized that the combination of nanobodies targeting these non-overlapping epitopes could potentially unlock potent anticancer activity. Consistent with our expectations, the nanobody-based antibodies (VHH-Fc) exhibited remarkable combined efficacy against the proliferation of HER2-positive cancer cells. Corroborating, A9B5-Fc, fashioned from a nanobody that binds to HER2 ECD I, showed superior synergistic inhibition efficacy, notably in cellular models demonstrating acquired resistance (**Figure 6**). Compared to conventional antibodies, nanobodies have a decreased immunogenicity owing to their smaller size and high solubility. Recent studies reported that a low immunogenicity risk profile was observed in patients receiving anti-HER2 nanobodies injection (40). In addition, the high affinity and specificity toward HER2 allow nanobodies to minimize the side effects and off-target effects. These results encourage us to explore potential clinical applications of A9B5-Fc in the future.

Unlike other HER receptors, HER2-ECD keeps an extended conformation with an exposed dimerization arm (DA) in ECD II (41). Thus, targeting epitopes of ECD II could block the HER2-containing dimerization. The suitable distance between the termini of ECD IV from two HER2 receptor molecules is a key pre-step to initiate signal transduction, revealing that anti-ECD4 is also a promising strategy (42–44). Despite ECD I and ECD III not being directly involved in HER2 activation, they are essential for the formation of binding pockets for DA of other HER2 partners (45, 46). Therefore, the functionality and mechanism of action (MOA) of HER2-targeted antibodies rely predominantly on their binding epitopes. Trastuzumab and pertuzumab, the two approved HER2-targeted antibodies, recognize ECD IV and II, respectively. They can inhibit the HER2-positive tumor cells by disrupting the ligand-independent or ligand-activated interaction between HER2 and HER3 (7, 30). Anti-ECD I or anti-ECD III antibodies have also been known to potentiate the reduction of HER2 functions *in vivo* and *in vitro (* 15, 47, 48). Notably, the antibody binding to ECD I demonstrates the potential to combat the trastuzumab-resistance (47, 49). Encouragingly, our research denotes that an anti-ECD I nanobody-based antibody, A9B5-Fc, had superior HER2 internalization activity and further mediated effective anti-proliferation effect, suggesting the theranostic potential for HER2-positive cancer treatment.

Receptor internalization is an important antitumor MOA of HER2-targeted antibodies. HER2 clustering induced by antibodies or their combinations can result in rapid HER2 internalization and inhibition of recycling, leading to surface HER2 downregulation and reduction of downstream phosphor-signaling (18, 31, 50). These inhibitory effects are able to translate into antiproliferative responses of anti-HER2 agents. We speculate that enhanced HER2 internalization mediated by A9B5-Fc contributes to its antitumor activity. It’s worth noting that not all anti-HER2 antibodies that promote receptor internalization possess inhibitory activity, some even demonstrate agonistic behavior (35). We observed that anti-ECD IV nanobody-based antibody, G1E4-Fc, mediated a substantial agonistic effect even at low nanomolar concentrations, despite its binding overlapping epitopes with trastuzumab (**Figures 3C**, **4B**). We speculate that G1E4-Fc might bind to specific sites, which help maintain suitable distance between ECD IV, leading to overactive HER2 downstream signaling pathways. Much more is required to explicate the underlying mechanisms.

It has been reported that the combination of antibodies targeting non-overlapping epitopes led to more effective anti-tumor activity in pre-clinical models and clinical trials (17, 18, 22, 36). The combination of trastuzumab and pertuzumab has been approved for the treatment of HER2-positive metastatic breast cancer. However, current combination therapy has not entirely surmounted the challenge of therapeutic resistance. Thus, to explore a powerful combination strategy, we examined the synergistic inhibitory efficiency of VHH-Fc mixtures or VHH-Fc plus approved monoclonal antibodies. Albeit VHH-Fc mixtures exerted limited combined efficacy, the collaboration of VHH-Fc with trastuzumab or pertuzumab showed an amplified synergistic inhibitory effect on tumor cell proliferation. Interestingly, both A9B5-Fc and A2G5-Fc, two anti-HER2-ECD I VHH-Fc, exhibited significantly superior synergistic activity than pertuzumab in trastuzumab combinations. Contrary to expectations, it was observed that the combination of A2G5-Fc and H2F5-Fc induced mild agonistic effects on HER2-positive tumor cells. This implies that the combination of no-competing antibodies may result in the loss of activity even generating counterproductive effects due to the convoluted multivalent binding of HER2 (51).

Ligand-driven HER2 heterodimerization, such as EGFR–HER2 and HER2–HER3, mediates compensatory signaling and has an important role in HER2-targeted therapy resistance (52). As such, it becomes imperative to discover efficacious drugs that can disrupt the crosstalk between HER2 and other EGFR family members. Pertuzumab, through binding to ECD II, can prevent HER2 heterodimerization, thereby diminishing HER2-driven signaling. Given that trastuzumab effectively obstructs cell proliferation in the absence of ligands, the complementary MOAs induced by the combination of these two approved antibodies offer enhanced antitumor potential, particularly in resistant carcinomas. In this work, we illustrated that A9B5-Fc exerts significantly stronger synergistic inhibitory activity with trastuzumab than pertuzumab in ligand-independent and ligand-driven tumor cells. This result suggests that the nanobody-based antibody targeting ECD I could provide adequate synergistic reduction for HER2 heterodimerization and homodimerization. This also proves the importance of targeting ECD I in blocking the functions of HER2 (47, 49).

It is well known that bispecific (biparatopic) antibodies may combine the MOA of two distinct antibodies, thereby potentially achieving elevated efficacy (31, 50, 53–58). Several preclinical studies claimed that biparatopic antibodies composed of fragments targeting ECD I and ECD IV demonstrated the superiority of combating resistance through multimodal MOA, including HER2 clustering, internalization, degradation, and trapped in a dimerization-incompetent state (59, 60). Based on these findings, we postulated that the construction of biparatopic antibodies derived from A9F5 and trastuzumab, with optimized formation, could lead to increased receptor internalization and heightened antitumor activity to surmount trastuzumab resistance.

The findings of this study have to be seen in light of some limitations. First, only two HER2-overexpressing tumor cell lines were used for functional analysis. The antitumor efficacy and MOAs of these nanobodies need to be further verified in more cell lines, including HER2-high, HER2-low, and HER2-negative tumor cells. Second, the synergistic activity of A9B5-Fc needs to be supported by further additional animal experiments.

## 5 Conclusion

Our findings indicate that the novel nanobody-based HER2-targeted antibody, A9B5-Fc, holds considerable potential as a robust synergistic collaborator with trastuzumab for trastuzumab-resistant tumors.

## Data availability statement

The raw data supporting the conclusions of this article will be made available by the authors, without undue reservation.

## Author contributions

XLL: Conceptualization, Formal Analysis, Funding acquisition, Methodology, Writing – original draft, Writing – review & editing. LL: Methodology, Writing – original draft. XL: Writing – review & editing. DJ: Methodology, Writing – review & editing. JD: Methodology, Writing – review & editing. JZX: Methodology, Writing – review & editing. YY: Methodology, Writing – review & editing. JYX: Methodology, Writing – review & editing. BC: Resources, Writing – review & editing. DX: Formal Analysis, Resources, Writing – review & editing. HH: Conceptualization, Formal Analysis, Funding acquisition, Project administration, Writing – review & editing.
