# Supplementary material for: A novel nanobody-based HER2-targeting antibody exhibits potent synergistic antitumor efficacy in trastuzumab-resistant cancer cells
Source: Front Immunol. 2023 Oct 25;14:1292839. doi: 10.3389/fimmu.2023.1292839 (PMC10634241; doi:10.3389/fimmu.2023.1292839)
Supplement: Supplementary file 1 [file Table_1.docx]

***Supplementary Material***

**A novel nanobody-based HER2-targeting antibody exhibits potent synergistic antitumor efficacy in trastuzumab-resistant cancer cells**

Xinlin Liu^1,2,*^, Linli Luan^3^, Xi Liu^4^, Dingwen Jiang^3^, Junwen Deng^1,2^, Jiazhen Xu^1,2^, Yang Yuan^1,2^, Jiyao Xing^1,2^, Bingguan Chen^3^, Dongming Xing^1,2,5,*^, Haiming Huang^3,*^

*Correspondence: lxl2021910024@qdu.edu.cn (X. Liu); haiming. huang@auambio.com (H. Huang); xdm@qdu.edu.cn (D. Xing).

**Supplementary Table 1. P-values for pairwise comparisions of HER2 internalization rates in SKBR3**

| Comparion | 0.5h | | 4h | |
| --- | --- | --- | --- | --- |
|  | Significant | P-value | Significant | P-value |
| A2G5-Fc vs. Trastuzumab | * | 0.0197 | ns | 0.7375 |
| A2G5-Fc vs. Pertuzumab | ** | 0.0011 | ns | 0.9986 |
| A9B5-Fc vs. Trastuzumab | *** | 0.0004 | **** | <0.0001 |
| A9B5-Fc vs. Pertuzumab | **** | <0.0001 | **** | <0.0001 |
| H2F5-Fc vs. Trastuzumab | ns | >0.9999 | ns | 0.0933 |
| H2F5-Fc vs. Pertuzumab | ns | 0.9201 | ns | 0.509 |
| G1E4-Fc vs. Trastuzumab | **** | <0.0001 | **** | <0.0001 |
| G1E4-Fc vs. Pertuzumab | **** | <0.0001 | **** | <0.0001 |

**Supplementary Table 2. P-values for pairwise comparisions of cell vialibity in NCI-N87**

| Comparion | Significant | P-value |
| --- | --- | --- |
| Trastuzumab vs. A2G5-Fc | * | 0.0145 |
| Trastuzumab vs. A9B5-Fc | *** | 0.0002 |
| Trastuzumab vs. H2F5-Fc | **** | <0.0001 |
| Trastuzumab vs. G1E4-Fc | **** | <0.0001 |

**Supplementary Table 3.** **P-values for pairwise comparisions of ligand-independent or ligand-dependent in NCI-N87**

| Comparion | None | | HRG | | EGF | |
| --- | --- | --- | --- | --- | --- | --- |
|  | Significant | P-value | Significant | P-value | Significant | P-value |
| Trastuzumab + Pertuzumab vs. A2G5-Fc + A9B5-Fc | *** | 0.0003 | * | 0.0189 | *** | 0.0001 |
| Trastuzumab + Pertuzumab vs. A2G5-Fc +H2F5-Fc | **** | <0.0001 | ns | 0.0768 | ns | 0.561 |
| Trastuzumab + Pertuzumab vs. A2G5-Fc + G1E4-Fc | *** | 0.0004 | * | 0.0452 | * | 0.0207 |
| Trastuzumab + Pertuzumab vs. A2G5-Fc + H2F5-Fc + G1E4-Fc | ** | 0.0045 | ns | 0.3983 | ns | 0.7697 |
| Trastuzumab + Pertuzumab vs. Trastuzumab | * | 0.0122 | *** | 0.0004 | **** | <0.0001 |
| Trastuzumab vs. A2G5-Fc + A9B5-Fc | ns | 0.5496 | ns | 0.2913 | ns | 0.9916 |
| Trastuzumab vs. A2G5-Fc +H2F5-Fc | *** | 0.0007 | ns | 0.109 | ** | 0.0013 |
| Trastuzumab vs. A2G5-Fc + G1E4-Fc | ns | 0.5845 | ns | 0.1646 | ns | 0.0974 |
| Trastuzumab vs. A2G5-Fc + H2F5-Fc + G1E4-Fc | ns | 0.9949 | * | 0.0183 | **** | <0.0001 |

**Supplementary Table 4. P-values for pairwise comparisions of ligand-independent in NCI-N87**

| Comparion | Significant | P-value |
| --- | --- | --- |
| Trastuzumab + Pertuzumab vs. A2G5-Fc -Fc +Trastuzumab | **** | <0.0001 |
| Trastuzumab + Pertuzumab vs. A2G5 -Fc +Pertuzumab | ns | 0.3672 |
| Trastuzumab + Pertuzumab vs. A9B5-Fc +Trastuzumab | **** | <0.0001 |
| Trastuzumab + Pertuzumab vs. A9B5-Fc +Pertuzumab | ns | 0.997 |
| Trastuzumab + Pertuzumab vs. H2F5-Fc +Trastuzumab | **** | <0.0001 |
| Trastuzumab + Pertuzumab vs. H2F5-Fc +Pertuzumab | ** | 0.004 |
| Trastuzumab + Pertuzumab vs. G1E4-Fc +Trastuzumab | **** | <0.0001 |
| Trastuzumab + Pertuzumab vs. G1E4-Fc + Pertuzumab | **** | <0.0001 |

**Supplementary Table 5. P-values for multiple** **comparisions of ligand-independent or ligand-dependent in NCI-N87**

| Comparion | None | | HRG | | EGF | |
| --- | --- | --- | --- | --- | --- | --- |
|  | Significant | P-value | Significant | P-value | Significant | P-value |
| Row 1 |  |  |  |  |  |  |
| Tra + Per vs. A2G5-Fc + Tra | **** | <0.0001 | * | 0.0257 | ** | 0.0017 |
| Tra + Per vs. A9B5-Fc + Tra | *** | 0.0003 | ns | 0.0633 | * | 0.0115 |
| Row 2 |  |  |  |  |  |  |
| Tra + Per vs. A2G5-Fc + Tra | **** | <0.0001 | ns | 0.5409 | **** | <0.0001 |
| Tra + Per vs. A9B5-Fc + Tra | ** | 0.0094 | ns | 0.1492 | **** | <0.0001 |
| Row 3 |  |  |  |  |  |  |
| Tra + Per vs. A2G5-Fc + Tra | *** | 0.0002 | ** | 0.0014 | ns | 0.069 |
| Tra + Per vs. A9B5-Fc + Tra | * | 0.0368 | *** | 0.0004 | ns | 0.1998 |
| Row 4 |  |  |  |  |  |  |
| Tra + Per vs. A2G5-Fc + Tra | * | 0.0113 | *** | 0.0004 | * | 0.0352 |
| Tra + Per vs. A9B5-Fc + Tra | ns | 0.0696 | * | 0.016 | ns | 0.1859 |
| Row 5 |  |  |  |  |  |  |
| Tra + Per vs. A2G5-Fc + Tra | ns | 0.4793 | ns | 0.8633 | ns | 0.0952 |
| Tra + Per vs. A9B5-Fc + Tra | ns | 0.9998 | ns | 0.0535 | ns | 0.3911 |
| Row 6 |  |  |  |  |  |  |
| Tra + Per vs. A2G5-Fc + Tra | ns | 0.5997 | ** | 0.0029 | ns | 0.6122 |
| Tra + Per vs. A9B5-Fc + Tra | ns | 0.9267 | * | 0.0272 | ns | 0.9645 |
| Row 7 |  |  |  |  |  |  |
| Tra + Per vs. A2G5-Fc + Tra | ns | 0.0652 | ns | 0.3753 | ns | 0.8881 |
| Tra + Per vs. A9B5-Fc + Tra | ns | 0.7242 | ns | 0.1289 | ns | 0.8538 |
| Row 8 |  |  |  |  |  |  |
| Tra + Per vs. A2G5-Fc + Tra | ns | 0.0563 | * | 0.0298 | ns | 0.1509 |
| Tra + Per vs. A9B5-Fc + Tra | ns | 0.8884 | ns | 0.7871 | ns | 0.512 |
